# Analysis of biochemical analytes using six sigma metrics with two analyzers at an Indian lab setting

**DOI:** 10.6026/973206300191043

**Published:** 2023-11-30

**Authors:** Ranjeeta Gadde, Venkatappa HM

**Affiliations:** 1 Kanva Diagnostic Services Private Ltd, #744, 11th Block, 2nd Stage, Marilingappa Extension, Nagarbhavi, Bengaluru - 560072, Karnataka, India

**Keywords:** Westgard rules, sigma metric, quality goal index

## Abstract

A zero defects goal was implemented in the clinical laboratory settings using a six-sigma model. Daily Internal Quality Control (IQC) and external quality control data from April-September 2023 was extracted to calculate the sigma metrics of 21 biochemical analytes based on Total Error Allowable (TEa), % bias and co-efficient of variation percent (CV%). A retrospective comparative study was conducted in the department of Clinical Biochemistry at Kanva Diagnostic Services Pvt. Ltd, Bengaluru, India. The analytical performance of the 21 biochemical analytes was tested on Cobas 6000 and C311 analyzers. Quality Goal Index (QGI) and root cause analysis was calculated to infer the reason for the deviation of six sigma. Method decision charts were plotted to show the comparison of the problem analytes on both the analyzers. On Cobas 6000 at level 1 IQC, out of 21 analytes, 10 analytes showed σ>6 and 10 analytes showed σ 3-6 and on C311, 15 analytes which showed σ>6 and 6 analytes that showed σ 3-6. On Cobas 6000 at level 2 IQC, out of 21 analytes, 12 analytes showed σ>6 and 8 analytes showed σ 3-6 and on C311 17 analytes showed σ>6 and 4 analytes showed σ 3-6. Creatinine failed to meet minimal sigma performance at both levels of IQC on Cobas 6000.

## Background:

Total Quality Management System in the diagnostic laboratory aims at the proper collection, analysis, and conveyance of precise and prompt reports to the right patient [1]. Clinical laboratory results play a significant role in decisions related to treatment of patients [2].The laboratory results if incorrect leads to serious complications like incorrect and delayed diagnosis and treatment [3,4]. The Total Testing Process (TTP) entails pre-analytical, analytical and post-analytical processes [5,6]. Analytical process is a dynamic area where maximum errors occurs leading to erroneous reports. Medical laboratories should strive to produce precise reproducible results as clinicians rely on these results for diagnosis, monitoring, and prognostication of patients [7,8]. Sigma (σ) metrics, a bench mark used in the field of quality management and quality control, particularly in healthcare and laboratory settings, to assess and monitor the performance and accuracy of diagnostic tests and measurement processes [9].Bill Smith of Motorola Corporation invented Six Sigma process [10] and Nevalainen *et al*. first applied the six sigma model, in medical laboratories [11]. These metrics help the clinical laboratories establish quality goals, ensure that their processes are meeting the required standards and make necessary corrections to maintain and enhance the quality of the patients' reports [12 ,13].The critical goal of sigma metrics is to implement risk management in the laboratory and to safeguard the patients [14,15].

As a predictor of risk, sigma metrics is a statistical measure of the capability of a process to produce results within predefined specifications or limits [16,17]. It's a way to assess how well a process is performing in terms of its ability to consistently produce output within acceptable quality boundaries. The exact number of errors in the analytical phase can be quantified only by Sigma metric and not by internal and external quality control data [18]. Therefore, it is of interest to compare the sigma metrics of 21 biochemical analytes on Cobas 6000 and Cobas C311, evaluate the root causes and take corrective action to improve the performance of the analytes with poor sigma metrics.

## Materials and methods:

A retrospective comparative study was conducted in the department of Clinical Biochemistry at Kanva Diagnostic Services Pvt. Ltd, Bengaluru. It is a standalone NABL accredited lab (MC-3756) which abides with the NABL guidelines and provides diagnostic services to around 400 outpatients daily. The equipment used for analysis was integrated modular analyzer Cobas 6000 and Cobas C311 (Roche Diagnostics, Mannheim, Germany). The data was extracted from consecutive runs of assay IQC samples for the 21 biochemical analytes over duration of six months from April to September 2023. The QC material used in the laboratory was a third party QC provided by BioRad which was received in lyophilized form and reconstituted by trained technical personnel. The IQC protocol was scheduled according to the NABL guidelines. Both physiological (Level 1) and pathological levels (Level 2) of QC were run daily before analyzing the patients' samples. The IQC data was monitored daily and the Levy-Jennings charts were interpreted using the standard Westgard rules (1_3S_/2_2S_/R_4S_/10x). The laboratory has enrolled in monthly EQAS program provided by BioRad. A stringent root-cause analysis was implemented followed by the needed corrective action for any deviations in IQC and EQAS results. Since the defects in the analytical performance cannot be assessed by IQC and EQAS results alone and hence, sigma metrics is required to quantify the exact number of defects in the testing process. The obtained sigma metric is inversely proportional to the quantitative defects.

## Statistical analysis

[1] Mean, SD and CV% were calculated for each analytes from the monthly IQC data.

[2] TEa values of the study analytes were taken from Clinical Laboratory Improvements Amendment (CLIA) 2024 and from Biological Variation (BV) database by Dr Carmen Ricos and colleagues available at www.westgard.com [19].

[3] The sigma metrics was calculated for all analytes using the above variables as mentioned below:

a. CV%: Standard-deviation/Mean x 100

b. Bias%: Lab EQAS result- Peer group mean / Peer group mean

c. Sigma Metric(σ) = TEa (%) - Bias(%)/CV%

[4] Sigma metric of > 6 is world class performance, σ value of 3-6 is good performance and σ value of <3 indicates poor performance of the test.

[5] QGI: % Bias/1.5 x CV%

[6] QGI<0.8 = Imprecision, QGI: 0.8-1.2 = Imprecision and Inaccuracy and QGI >1.2 = Inaccuracy [20].

[7] Statistical software: The obtained data was entered in Microsoft Excel Version 16 and the histograms were plotted.

[8] The normalized sigma method decision charts were extracted from the website https://www.westgard.com/normalized-opspecs-calculator.htm. Parameters such as TEa, bias% and CV% were inputted and the graph was plotted with bias% on y-axis and imprecision on x-axis. Sigma metric zones are presented on the Sigma method decision charts i.e., the zone closest to the graph's origin 'World class performance' is 6σ zone, followed by the 5σ 'Excellent' zone, 4σ 'Good' zone, 3σ 'Marginal' zone, 2σ 'Poor' zone and the remaining portion of the chart is marked as unacceptable.

## Results:

The current study evaluated the sigma metrics for 21 biochemical analytes run on Cobas 6000 and Cobas C311. The comparison of CV% of level 1 IQC for the biochemical on Cobas 6000 and Cobas C311 from the month of April to September 2023 are tabulated in Table 1. The comparison CV% of level 2 IQC for the biochemical analytes on Cobas 6000 and Cobas C311 from the month of April to September 2023 are emphasized in Table 2. The comparison the bias % for the biochemical analytes on Cobas 6000 and Cobas C311 from the month of April to September 2023 are displayed Table 3. The comparison of the Sigma metrics and QGI for biochemical analytes from the month of April to September 2023 are shown in Table 4. The performance of the 21 biochemical analytes on Sigma metrics scale are categorized into three levels i.e., >6,3-6 and <3 as summarized in Table 5. QGI for creatinine for level 1 IQC was 0.25 and for level 2 IQC was 0.24, which indicated imprecision in the QC values.

The method decision charts were plotted for level 1 and 2 IQC for Creatinine on Cobas C311 and Cobas 6000. It shows that on Cobas C311 Creatinine was near 4σ zone and on Cobas 6000 near 2σ zone farthest from the origin indicating lowest sigma value.

## Discussion:

**Figure 1:**
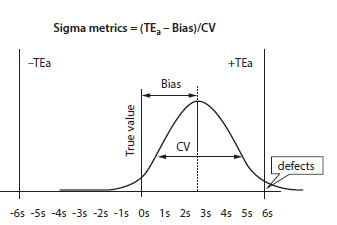
Graphic description of the working of Sigma metrics equation.

In the current study, the analytical performance of 21 biochemical analytes was compared on two automated analyzers, Cobas 6000 and Cobas C311. The sigma metrics was effectively evaluated for every analyte based on the IQC and EQAS data obtained from April 2023-September 2023. In clinical laboratories since the reliability of test reports relies on accuracy and precision, QGI was calculated for the analyte with σ<3 to reveal the accuracy and precision. The CV% measures variability and random error and Bias% which indicate accuracy and systemic errors in the testing process. TEa targets were derived from CLIA'2024 and BV data. The graphic description on the working of the sigma metrics equation is depicted in Figure 1.

**Figure 2:**
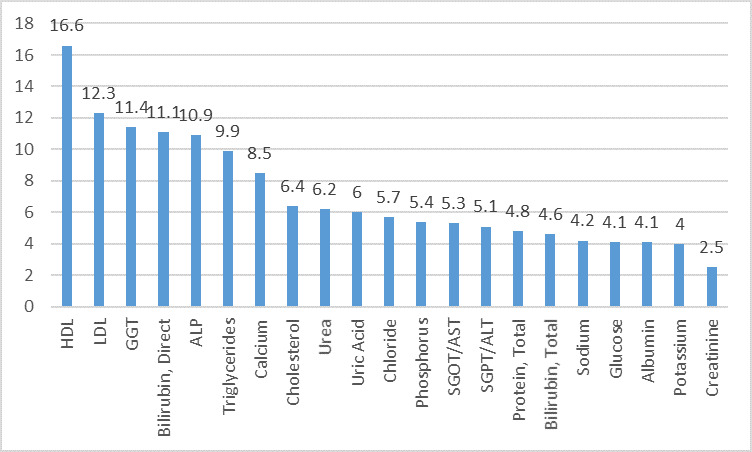
Sigma metrics for biochemical analytes on Cobas 6000 level 1 IQC in descending order.

On Cobas 6000 at level 1 IQC, out of 21 analytes, 10 analytes which showed world class performance (σ>6) were Alkaline Phosphatase (ALP), Bilirubin Direct, Calcium, Gamma Glutamyl Transferase (GGT), Low Density Lipoprotein (LDL), Triglycerides, Uric acid, Urea, Cholesterol and High-Density Lipoprotein (HDL). The other 10 analyzes which showed good performance (σ3-6) were Albumin, Serum Glutamyl Pyruvate Transferase(SGPT), Serum Glutamyl Oxaloacetate Transferase (SGOT), Bilirubin Total, Glucose, Phosphorus, Protein Total, Sodium, Potassium and Chloride. Only Creatinine showed poor performance (σ<3) as depicted in Figure 2.

**Figure 3:**
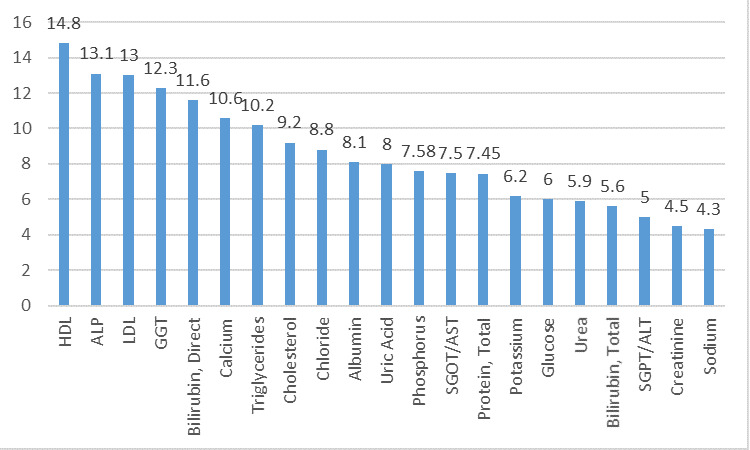
Sigma metrics for biochemical analytes on Cobas C311 level 1 IQC in descending order.

On Cobas C311 at level 1 IQC, out of 21 analytes, 15 analytes which showed world class performance (σ>6) were Albumin, ALP, SGOT, Bilirubin Direct, Calcium, GGT, LDL, Phosphorus, Protein Total, Triglycerides, Uric acid, Cholesterol, HDL, Potassium and Chloride. The 6 analytes that showed good performance (σ3-6) were SGPT, Bilirubin Total, Creatinine, Glucose, Urea and Sodium as shown in Figure 3. All analytes showed good performance.

**Figure 4:**
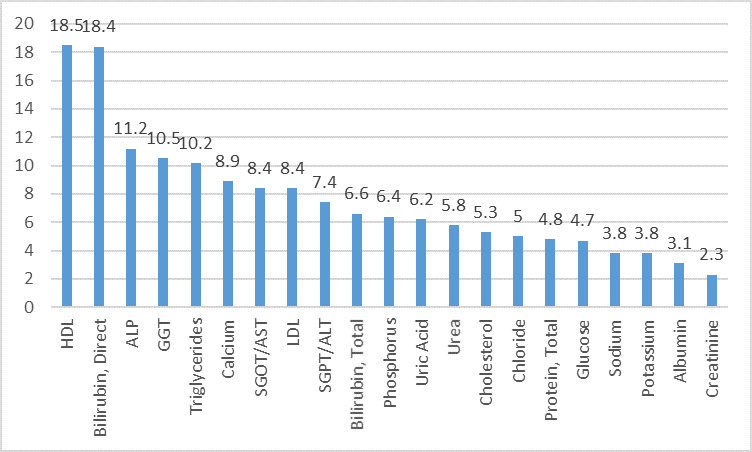
Sigma metrics for biochemical analytes on Cobas 6000 level 2 IQC in descending order

On Cobas 6000 at level 2 IQC, out of 21 analytes, 12 analytes which showed world class performance (σ>6) ALP, SGPT, SGOT, Bilirubin Direct, Bilirubin Total, Calcium, GGT, LDL, Phosphorus, Triglycerides, Uric acid and HDL. The 8 analytes that showed good performance (σ3-6) were Albumin, Glucose, Protein Total, Urea, Cholesterol, Sodium, Potassium and Chloride. Only Creatinine showed poor performance (σ<3) as depicted in Figure 4.

**Figure 5:**
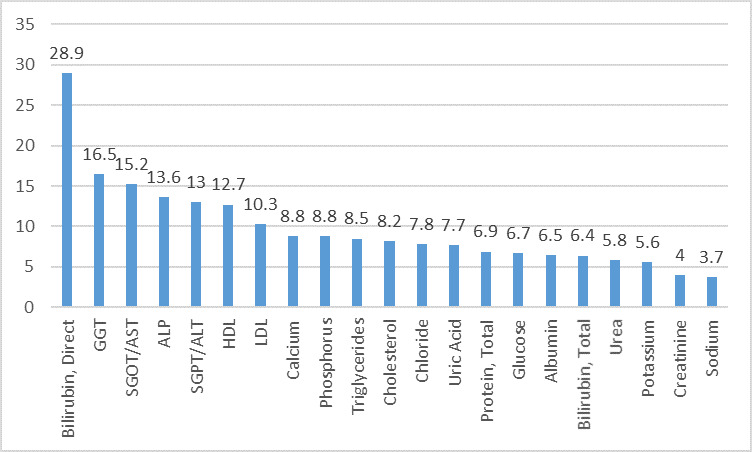
Sigma metrics for biochemical analytes on Cobas C311 level 2 IQC in descending order.

On Cobas C311 at level 2 IQC, out of 21 biochemical analytes, 17 analytes which showed world class performance (σ>6) were Albumin, ALP, SGPT, SGOT, Bilirubin Direct, Bilirubin Total, Calcium, GGT, Glucose, LDL, Phosphorus, Protein total, Triglycerides, Uric acid, Cholesterol, HDL, and Chloride. The 4 analytes that showed good performance (σ3-6) were Creatinine, Urea, Sodium and Potassium as represented in Figure 5.

**Figure 6:**
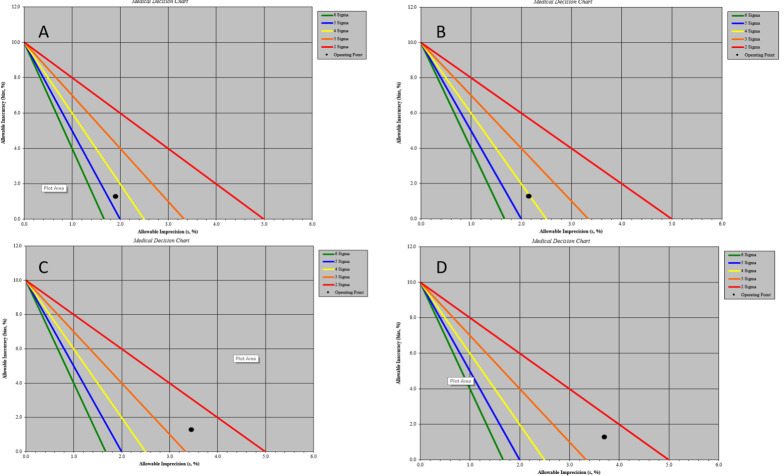
Sigma method decision chart showing Inaccuracy (bias%) is on y-axis and Imprecision (CV%) is on x-axis. A: Sigma metric 4.5 for level 1 QC for Creatinine on Cobas C311;B: Sigma metric 4.0 for level 2 QC for Creatinine on Cobas C311; C: Sigma metric 2.5 for level 1 QC for Creatinine on Cobas 6000; D: Sigma metric 2.3 for level 2 QC for Creatinine on Cobas 6000.

Creatinine failed to meet minimal sigma performance at both levels of IQC on Cobas 6000. QGI calculated and root cause analysis for Creatinine showed imprecision. The corrective action to be adopted was an additional QC rule 4_1s_ apart from 1_3S_ /2_2S_/R_4S_ /10_x_ and frequent calibration of Creatinine. Figure 6 demonstrates the method decision charts plotted for the performance of Creatinine on Cobas 6000 and Cobas C311. The factors such as reconstitution of controls and calibrators, stability of controls and calibrators, equipment maintenance, scheduled preventive maintenance, quality of water used in analysis, on board stability of reagents, inadequate sample aspiration, improper mixing, contamination in sample cuvettes and training of the personnel were all taken care.

Similar results were reported in a retrospective observational study conducted in clinical Biochemistry laboratory in KR hospital, Mysuru. Sigma metrics was calculated for renal function tests and electrolyte parameters which were analyzed on Cobas 6000. The study reported poor performance with σ<3 for Creatinine in level 1 and 2 IQC [21]. An observational study conducted in Ethiopian Public Health Institute (EPHI) clinical chemistry reference laboratory tested 18 biochemical analytes on Cobas 6000. The results showed consensus with the results of the current study about Creatinine for both the levels of control. Unlike the findings of our study, low sigma metrics were reported for Urea and Chloride [3].

A study in Beijing hospital was conducted under national creatinine trueness verification scheme. They used two different concentration levels of fresh frozen serum for evaluation of Creatinine measurement on automated analyzer Roche. The results showed that there was a requisite for 7- 45.1% of the laboratories to improve their measurement procedures for enzymatic method. 11.5-73 % of the laboratories must try to improve the trueness for Jaffe's method. 3.1-5.3% of the laboratories ought to emphasize on both precision and trueness. The results of this study revealed poor performance for creatinine which is analogous to our study [22].

A retrospective study in Clinical Laboratory of Hunan Provincial People's Hospital, China was performed using Beckman Coulter AU5800 analyzer and compared two level IQC sigma values of 19 biochemical analytes. Out of which 10 analytes showed good performance on sigma metric scale and 9 analytes showed a sigma value of <4. QGI analysis and RCA further indicated inaccuracy and imprecision. These findings were in consensus with the results of our study which showed imprecision for Creatinine at both levels IQC [23].

A retrospective study conducted in Turkey, calculated six sigma values for 21 routine biochemistry parameters using Cobas c702. A three-month IQC data was taken for the calculation of CV%. The results of this study were in consensus with the results of our study with respect to creatinine i.e., according to CLIA goals the sigma metrics was <3 which showed imprecision at level 1IQC [24].

Contradictory results were reported in a retrospective study followed by prospective study conducted in clinical Biochemistry department in JSS Medical College, Mysuru. The IQC data for 31 analytes was collected from Cobas 6000 and e411 retrospectively followed by prospective study for analytes which showed σ<2. In contrast to our study, Creatinine showed world class performance (σ = 6.39) in level 1 internal QC and good performance in level 2 IQC (σ = 5.34), Sodium and Potassium showed poor performance (σ<2) [25].

Contradicting with the results of the current study, a prospective study conducted in King Abdulaziz Specialist Hospital, Sakaka, evaluated the sigma metrics for 25 biochemical parameters on Cobas 6000. The performance of Creatinine (σ=4.66 at level 1 and σ=5.06 at level 2 IQC) and Sodium, Potassium and Chloride (σ<3) were not in agreement with the results of the current study [26].

A retrospective observational study in Indonesia showed that sigma metrics calculated for 11 biochemical analytes on Cobas C311 showed good performance for Creatinine in accordance with the findings of the current study. However, urea showed unacceptable performance which was not in agreement with the findings of our study [27].

## Conclusion:

The current study evaluated the performance of 21 biochemical analytes by sigma metrics on two automated analyzers Cobas 600 and C311. The study focused on to spot the parameter which deviated from the six sigma scale. The only biochemical analyte that showed poor performance on Cobas 6000 at both levels of IQC was Creatinine. The problem-solving strategy for the imprecision shown by Creatinine included following stringent quality control rules and frequent calibration.

## Funding:

No additional funding was required.

## Ethics approval:

Ethical approval was not required as this study was based on retrospective data available from the laboratory.

## Figures and Tables

**Table 1:** Comparison of CV% of level 1 control for biochemical analytes on Cobas 6000 and Cobas C311 from April-September 2023

**Parameter**	**April**		**May**		**June**		**July**		**Aug**		**Sept**		**Average**	
	**A**	**B**	**A**	**B**	**A**	**B**	**A**	**B**	**A**	**B**	**A**	**B**	**A**	**B**
Albumin	1.18	0.94	1.43	1.14	1.64	1.49	1.89	1.17	2.1	0.78	2.57	0.99	1.8	1.08
ALP	2.1	2.08	1.49	0.9	1.49	1.33	1.18	2.35	3.04	1.8	1.53	1.1	1.8	1.59
SGPT	2.9	2.51	2.81	3.1	2.82	3.08	4.51	3.83	3.25	3.23	3.28	2.53	3.26	3.04
SGOT	3.15	2.32	3.23	2.12	2.93	2.27	2.73	2.26	3.15	2.59	2.11	1.71	2.88	2.21
Bilirubin, Direct	4.2	2.92	3.75	3.61	4.5	3.79	3.81	5.13	4.78	4.46	3.85	3.71	4.14	3.93
Bilirubin, Total	4.21	2.98	4.5	1.99	2.63	2.79	3.37	4.29	4.63	4.1	3.81	3.1	3.85	3.2
Calcium	1.14	1	1.36	0.84	1.15	0.74	1.15	0.91	1.35	0.85	1.44	1.37	1.26	0.95
Creatinine	3.1	1.06	4.45	3.25	3.33	1.79	3.12	1.3	3.3	1.81	3.48	2.36	3.46	1.92
GGT	1.74	1.59	1.12	0.94	1.13	1.1	1.44	1.11	0.97	0.96	1.48	1.27	1.31	1.16
Glucose	2.21	1.11	2.25	0.86	1.54	1.49	1.4	1.26	1.64	1.13	1.26	1.38	1.71	1.2
LDL	1.37	1.04	1.53	1.31	1.38	2.7	1.17	0.86	1.66	1.95	2.57	1.19	1.61	1.5
Phosphorus	2.22	1.26	2.2	1.02	1.91	1.89	1.61	0.77	1.6	0.95	1.32	1.08	1.81	1.16
Protein, Total	1.4	1	1.71	1.14	1.24	1.21	1.56	0.92	1.7	0.86	1.38	1.2	1.49	1.05
Triglycerides	1.98	1.36	1.41	1.15	1.72	1.74	1.38	1.07	1.37	1.78	1.72	1.66	1.59	1.46
Uric Acid	2.14	2.08	1.5	0.85	1.75	1.07	2.17	1.47	1.94	1.27	2.14	1.26	1.94	1.33
Urea	2.15	3.05	1.53	1.02	1.54	1.48	1.81	1.21	1.81	1.48	1.75	1.69	1.765	1.65
Cholesterol	1.5	1.38	1.22	0.89	1.36	1.28	1.36	1.07	1.32	0.85	2.07	1.35	1.47	1.13
HDL	0.96	1.19	1.75	1.6	1.24	2.38	1.72	1.3	1.09	1.51	1.39	1.41	1.35	1.56
Sodium	1.08	0.8	1.01	0.74	0.8	0.54	0.81	0.67	0.67	0.6	0.93	0.53	0.88	0.64
Potassium	1.43	0.62	1.32	0.95	0.9	0.54	1.05	0.48	1.13	0.76	1.22	0.71	1.17	0.67
Chloride	0.97	0.56	1.27	0.74	0.97	0.43	0.81	0.77	0.94	0.42	1.13	0.72	1.01	0.6
**Note:**
A = Cobas 6000
B= Cobas C311
SGPT = Serum Glutamic Pyruvic Transaminase
SGOT= Serum Glutamic Oxaloacetic Transaminase
GGT = Gamma Glutamyl Transferase
LDL= Low Density Lipoprotein
HDL= High Density Lipoprotein

**Table 2:** Comparison of CV% of level 2 control for biochemical analytes on Cobas 6000 and Cobas C311 from April-September 2023

**Parameter**	April		**May**		**June**		**July**		**Aug**		**Sept**		**Average**	
	**A**	B	**A**	**B**	**A**	**B**	**A**	**B**	A	**B**	**A**	**B**	**A**	**B**
Albumin	2.76	1.14	2.43	1.59	2.08	1.32	2.75	1.43	2.8	1.01	3.91	1.51	2.78	1.33
ALP	1.65	1.91	2.08	0.88	1.37	1.31	1.66	2.2	2.46	2.12	1.92	0.8	1.85	1.53
SGPT/ALT	1.85	1.13	1.38	0.8	2.24	1.18	1.91	0.93	3.27	2.01	1.82	0.99	2.07	1.17
SGOT/AST	2.5	1.2	1.24	0.84	2.32	0.99	1.7	0.72	2.28	1.68	1.81	1.11	1.97	1.09
Bilirubin, Direct	2.79	1.84	2.4	1.4	3.05	1.73	1.95	0.95	2.3	1.53	2.51	2.08	2.5	1.58
Bilirubin, Total	2.41	3.33	3.18	1.26	1.91	2.26	2	2.16	3.9	3.06	2.94	4.69	2.72	2.79
Calcium	1.27	1.16	1.28	1.12	1.26	0.57	1.2	0.69	1.05	2.2	1.19	1.14	1.2	1.14
Creatinine	4.06	1.96	3.78	3.49	4.09	2.1	3.35	1.75	3.27	1.76	3.71	1.94	3.71	2.16
GGT	1.76	1.67	0.68	0.79	1.61	0.61	1.86	0.73	1.05	0.74	1.56	0.67	1.42	0.86
Glucose	1.63	1.07	1.74	1.1	1.34	0.93	1.41	0.9	1.92	1.39	0.98	1.16	1.5	1.09
LDL	2.33	2.08	2.13	1.71	2.21	1.73	2.54	1.53	1.97	2.71	3.09	1.67	2.37	1.9
Phosphorus	1.53	1.07	2.08	0.99	1.8	1.47	1.49	0.83	1.2	0.86	1.06	0.75	1.52	0.99
Protein, Total	2.08	1.37	1.86	1.1	1.4	1.12	0.93	0.96	1.62	1.03	1.15	1.15	1.5	1.12
Triglycerides	1.39	1.93	1.39	1.97	1.61	1.43	1.51	1.88	1.73	1.66	1.62	1.64	1.54	1.75
Uric Acid	1.68	2.48	1.68	0.94	1.51	1.17	2.36	1.36	2.14	1.47	1.91	0.94	1.88	1.39
Urea	1.99	3.43	1.84	1.2	1.85	1.12	2.13	1.68	1.89	1.45	1.65	1.09	1.89	1.66
Cholesterol	1.4	1.41	1.58	1.32	1.85	0.92	1.92	1.32	1.82	1.52	2.08	1.15	1.77	1.27
HDL	2.48	1.47	2.48	2.14	0	2.25	1.18	1.61	0	1.58	1.17	1.81	1.21	1.81
Sodium	1.12	0.95	1.12	0.71	0.87	0.71	0.79	0.67	0.71	0.71	1.25	0.71	0.97	0.74
Potassium	1.54	1.32	1.31	0.72	1.1	0.45	0.9	0.61	0.88	0.67	1.79	0.75	1.25	0.75
Chloride	1.25	1.15	1.22	0.6	1.29	0.57	1.15	0.48	1.05	0.72	1.01	0.59	2.78	0.68
**Note:**
A = Cobas 6000
B= Cobas C311
SGPT = Serum Glutamic Pyruvic Transaminase
SGOT= Serum Glutamic Oxaloacetic Transaminase
GGT = Gamma Glutamyl Transferase
LDL= Low Density Lipoprotein
HDL= High Density Lipoprotein

**Table 3:** Comparison the Bias % for the biochemical analytes on Cobas 6000 and Cobas C311 from the month of April to September 2023

**Parameter**	**April**		**May**		**June**		**July**		**Aug**		**Sept**		**Average**	
	**A**	**B**	**A**	**B**	**A**	**B**	**A**	**B**	**A**	**B**	**A**	**B**	**A**	**B**
Albumin	-0.2	-1.83	1.62	-2.43	2.23	-0.95	1.91	1.91	-0.47	0	-2.16	-1.37	0.48	-0.77
ALP	-0.14	1.28	0.84	-1.27	1.08	-2.44	1.77	-0.59	0	0	-1.82	-2.28	0.28	-0.88
SGPT/ALT	-5.6	-3.01	2.18	2.18	2.11	0.49	-1.7	0.85	-3.52	0	-4.47	-2.43	-1.83	-0.32
SGOT/AST	-6.06	-7.57	1.97	-1.31	1.04	0.58	0	-1.29	-1.02	-0.76	1.08	0.36	-0.49	-1.66
Bilirubin, Direct	0.35	0.35	-1.09	0	-2.61	0	0	-1.31	-4.18	-3.25	-2.55	-3.64	-1.68	-1.3
Bilirubin, Total	1.04	1.7	6.79	3.88	0.36	-1.1	2.38	4.77	-0.36	1.28	1.56	1.69	1.96	2.03
Calcium	-1.27	0	-1.02	0.17	1.89	0.71	-1.67	-0.83	-1.63	0	5.12	5.12	0.23	0.86
Creatinine	0.98	1.96	1.58	0.52	-0.76	-1.68	1.63	1.63	1.57	2.1	3.09	3.09	1.34	1.27
GGT	1.35	-0.27	1.82	2.73	0	1.29	-0.63	1.27	-1.42	-0.35	-1.04	-0.25	0.01	0.73
Glucose	3.7	0	-0.68	0.68	-0.87	0	1.06	1.82	2.72	1.67	-0.76	0	0.86	0.69
LDL	0	0.43	-0.34	3.09	1.96	1.47	0.99	-0.99	0.6	1.02	-2.66	-2.66	0.09	0.39
Phosphorus	-3.55	2.03	1.22	1.89	-0.13	1.24	0.55	1.39	1.63	0.65	1.2	0	0.15	1.2
Protein, Total	0.52	0.26	-0.53	0	1.26	-0.84	0.62	0.62	0.93	0	1.65	1.01	0.74	0.17
Triglycerides	-1.98	-1.48	0.19	1.37	-1.37	-0.31	-1	-0.1	-0.6	0	-0.45	0.91	-0.86	0.06
Uric Acid	0.69	0.69	0	0.3	-2.62	-0.12	-2.7	-1.35	-6.5	-4.47	0.35	0.35	-1.79	-0.76
Urea	-3.54	0	-3.54	-1.41	0.54	1.35	-1.57	-0.52	-4.38	-4.13	0	0	-2.08	-0.78
Cholesterol	-1.42	-0.56	2.94	-1.26	4.93	0	-2.45	-1.22	-1.49	-0.74	0.78	0.78	0.54	-0.5
HDL	-3.95	-3.95	-2.7	-3.24	2.94	-3.22	-3.9	-3.9	-2.71	-1.58	-4.58	-2.75	-2.48	-3.1
Sodium	-0.64	0.64	-0.89	0.89	0.8	1.61	0.76	1.53	0	0.67	1.61	1.84	0.27	1.19
Potassium	-1.73	0.57	0.49	0.46	1.71	1.52	-0.34	0.34	-0.21	0.86	1.61	0.96	0.25	0.78
Chloride	-0.89	0	-1.94	-0.12	-1.09	-1.7	-0.8	0.34	-1.98	-1.38	1.75	0.87	-0.82	-0.33
**Note:**
A = Cobas 6000
B= Cobas C311
SGPT = Serum Glutamic Pyruvic Transaminase
SGOT= Serum Glutamic Oxaloacetic Transaminase
GGT = Gamma Glutamyl Transferase
LDL= Low Density Lipoprotein
HDL= High Density Lipoprotein

**Table 4:** Comparison of the sigma metrics for biochemical analytes on Cobas 6000 and Cobas C311 from the month of April to September 2023

**Parameter**	**CV% Level 1**		**CV% Level 2**		**% Bias**		**% Tea CLIA**	**Sigma Metrics Level 1**		**Sigma Metrics Level 2**	
	**A**	**B**	**A**	**B**	**A**	**B**		**A**	**B**	**A**	**B**
Albumin	1.8	1.08	2.78	1.33	0.48	-0.77	8	4.1	8.1	3.1	6.5
ALP	1.8	1.59	1.85	1.53	0.28	-0.88	20	10.9	13.1	11.2	13.6
SGPT/ALT	3.26	3.04	2.07	1.17	-1.83	-0.32	15	5.1	5	7.4	13
SGOT/AST	2.88	2.21	1.97	1.09	-0.49	-1.66	15	5.3	7.5	8.4	15.2
Bilirubin, Direct	4.14	3.93	2.5	1.58	-1.68	-1.3	44.5 BV	11.1	11.6	18.4	28.9
Bilirubin, Total	3.85	3.2	2.72	2.79	1.96	2.03	20	4.6	5.6	6.6	6.4
Calcium	1.26	0.95	1.2	1.14	0.23	0.86	11	8.5	10.6	8.9	8.8
Creatinine*	3.46	1.92	3.71	2.16	1.34	1.27	10	2.5*	4.5	2.3*	4
GGT	1.31	1.16	1.42	0.86	0.01	0.73	15	11.4	12.3	10.5	16.5
Glucose	1.71	1.2	1.5	1.09	0.86	0.69	8	4.1	6	4.7	6.7
LDL	1.61	1.5	2.37	1.9	0.09	0.39	20	12.3	13	8.4	10.3
Phosphorus	1.81	1.16	1.52	0.99	0.15	1.2	10	5.4	7.58	6.4	8.8
Protein, Total	1.49	1.05	1.5	1.12	0.74	0.17	8	4.8	7.45	4.8	6.9
Triglycerides	1.59	1.46	1.54	1.75	-0.86	0.06	15	9.9	10.2	10.2	8.5
Uric Acid	1.94	1.33	1.88	1.39	-1.79	-0.76	10	6	8	6.2	7.7
Urea	1.765	1.65	1.89	1.66	-2.08	-0.78	9	6.2	5.9	5.8	5.8
Cholesterol	1.47	1.13	1.77	1.27	0.54	-0.5	10	6.4	9.2	5.3	8.2
HDL	1.35	1.56	1.21	1.81	-2.48	-3.1	20	16.6	14.8	18.5	12.7
Sodium	0.88	0.64	0.97	0.74	0.27	1.19	4	4.2	4.3	3.8	3.7
Potassium	1.17	0.67	1.25	0.75	0.25	0.78	5	4	6.2	3.8	5.6
Chloride	1.01	0.6	2.78	0.68	-0.82	-0.33	5	5.7	8.8	5	7.8
**Note:**
A = Cobas 6000
B= Cobas C311
CLIA= Clinical Laboratory Improvement Amendments
BV= Biological Variation
SGPT = Serum Glutamic Pyruvic Transaminase
SGOT= Serum Glutamic Oxaloacetic Transaminase
GGT = Gamma Glutamyl Transferase
LDL= Low Density Lipoprotein
HDL= High Density Lipoprotein
*Creatinine showed σ<3

**Table 5:** Sigma metrics for level 1 and level 2 IQC for the biochemical analytes on Cobas 6000 and Cobas C311

	**Cobas 6000**	**Cobas C311**	**Cobas 6000**	**Cobas C311**
**Sigma Metrics**	**Level 1**	**Level 1**	**Level 2**	**Level 2**
σ>6	ALP, Bilirubin Direct, Calcium, GGT, LDL, Triglycerides, Uric acid, Urea, Cholesterol, HDL	Albumin, ALP, SGOT, GGT Bilirubin Direct, Calcium, LDL, HDL, Chloride, Phosphorus,Protein Total, Triglycerides, Uric acid, Cholesterol,Potassium	ALP, SGOT, SGPT,Bilirubin Direct, Bilirubin Total,Calcium, GGT,LDL, Phosphorus, Triglycerides, Uric acid, Cholesterol	Albumin, ALP, SGOT, SGPT,Bilirubin Direct, Bilirubin Total,Calcium,GGT, Glucose,LDL, Phosphorus, Protein Total, Triglycerides, Uric acid, Cholesterol,HDL, Chloride
σ3-6	Albumin, SGPT, SGOT, Bilirubin Total, Glucose, Phosphorus,Protein Total, Sodium, Potassium,Chloride	SGPT, Urea,Bilirubin Total,Creatinine,Glucose,Sodium	Albumin, Urea, Glucose,Protein Total, Cholesterol,Sodium, Potassium,Chloride	Urea,Creatinine,Sodium, Potassium
σ<3	Creatinine		Creatinine	
